# T-cell lymphoma with POEMS syndrome

**DOI:** 10.3892/ol.2014.2810

**Published:** 2014-12-17

**Authors:** FANGWEN ZOU, ZHENHUA LI, JIN-AN MA, ZHENHUA QIU, YI-FANG TANG, JIAO-YUN ZHENG

**Affiliations:** 1 Department of Oncology, Xiangya Second Hospital of Center South University, Changsha, Hunan 410011, P.R. China; 2 Department of Pathology, Xiangya Second Hospital of Center South University, Changsha, Hunan 410011, P.R. China

**Keywords:** angioimmunoblastic T-cell lymphoma, POEMS syndrome

## Abstract

Angioimmunoblastic T-cell lymphoma (AITL) is a unique subtype of peripheral T-cell lymphoma. POEMS syndrome is a rare paraneoplastic syndrome caused by an underlying plasma cell disorder (PCD). The occurrence of AITL with POEMS syndrome has rarely been reported in the literature. The current study presents the case of a 53-year-old male who presented with a rapidly proliferative lymph node on the left neck, which was identified as an AITL on biopsy. The patient also exhibited the complications of polyneuropathy, M-proteinemia, hepatosplenomegaly, left ventricular hypertrophy, endocrinopathy and skin changes, and was therefore diagnosed with POEMS syndrome. To the best of our knowledge, the present study is the first to report a case of AITL with POEMS syndrome. The findings in this case suggest that the aberrant clones of B cells can also be caused by AITL.

## Introduction

AITL is a rare and unique subtype of peripheral T-cell lymphoma, accounting for 2–5% of all non-Hodgkin lymphomas (1). POEMS syndrome is a rare paraneoplastic syndrome secondary to a plasma cell dyscrasia. The POEMS acronym, which was coined by Bardwich *et al* (2) in 1980, refers to the features of the syndrome: Polyneuropathy (P), organomegaly (O), endocrinology (E), monoclonal protein (M), skin changes (S), which combine with a range of other clinical and pathological symptoms, including fever, cachexia, edema, thrombocytosis and multicentric Castleman’s disease. The current study presents a rare case of AITL with POEMS syndrome and reviews the literature on AITL. Written informed consent was obtained from the patient.

## Case report

**Figure 1:**
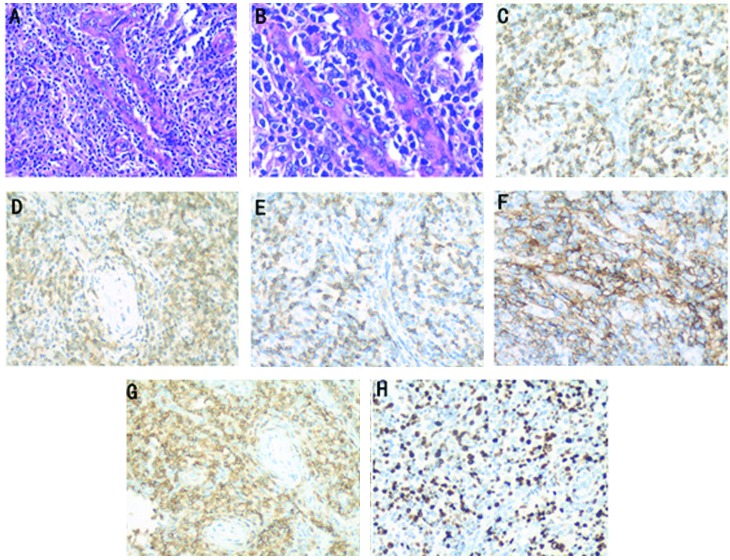
Histological analysis of the left neck lymph nodes showing (A) loss of the normal architecture, except for the presence of occasional depleted follicles with concentrically arranged follicular dendritic cells, and effacement of the architecture by polymorphic infiltrate with marked vascular proliferation [hematoxylin and esoin (HE) staining; magnification, ×200]. (B) Higher magnification of the image in (A) (HE staining; magnification, ×400). Immunohistochemical staining showed positive expression of (C) cluster of differentiation (CD)3 (+), (D) CD4 (+), (E) CD8 (+), (F) CD21 (++), (G) CD45RO (++) and (H) Ki-67 (++; 60%).

**Figure 2:**
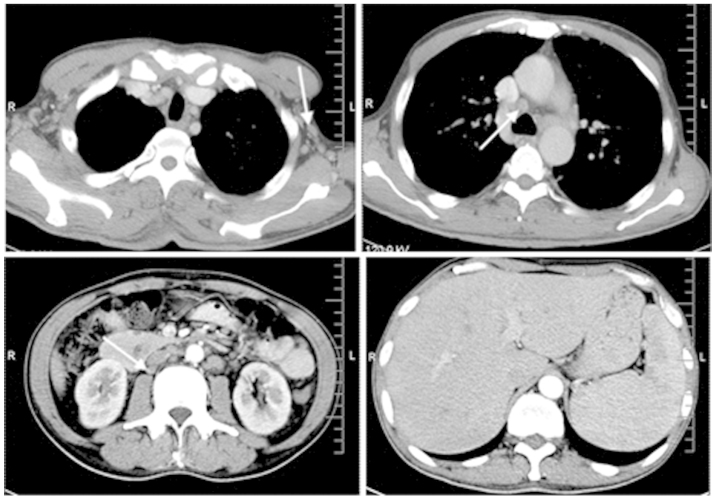
Computed tomography scans revealing swelling of the axillary (top left image), mediastinal (top right image) and retroperitoneal (bottom left image) lymph nodes, and hepatosplenomegaly (bottom right image). Arrows indicate the areas of swelling.

A 53-year-old male developed weakness of the lower limbs, numbness and stabbing pains below the ankle and wrist joints, accompanied by a low fever and night sweating. One month later, the patient developed a red, full-body rash and noticed gradually worsening instability when walking. A rapidly proliferative painless lymph node on the left side of the neck was also noted. As a consequence, the patient was admitted to the Xiangya Second Hospital of Center South University (Changsha, Hunan, China). A physical examination revealed lower limb paresis (grade 4/5 according to the manual muscle testing) (3), a high fever, a red rash and swelling of the left neck lymph nodes to a size of 2.0×1.5 cm^2^. Blood analysis revealed the following results: White blood cell count, 14.6×10^9^/l (normal range, 3.0–10.0×10^9^/l) [87.6% neutrophils (normal range, 50–70%) and 5.6% lymphocytes (normal range, 20–40%)]; hemoglobin, 88 g/l (normal range, 20–160 g/l); platelet count, 462×10^9^/l (normal range, 100–300×10^9^/l); sodium, 133 mmol/l (normal range, 136–146 mmol/l); calcium, 1.96 mmol/l (normal range, 2.08–2.80 mmol/l); albumin, 22.8 g/l (normal range, 35–50 g/l); lactate dehydrogenase, 290 IU/l (normal range, 100–300 IU/l); rheumatoid factors, 23.7 IU/ml (normal range, 0.0–20.0 IU/l); erythrocyte sedimentation rate, 65 mm/h (normal range, 0–15 mm/h); and C-reactive protein, 15 mg/dl (normal range, 0–10 mg/dl). Endocrine investigations revealed reduced levels of neo-hombreol F (130 ng/dl; normal range, 250–836 ng/dl). The serum immunoglobulin (Ig)G (31.5 g/l), IgA (8.55 g/l) and IgM (3.12 g/l) levels were all high (normal ranges, 7.6–166 g/l, 0.71–3.35 g/l and 0.48–2.12 g/l, respectively), and immunoelectrophoresis revealed monoclonal gammopathy (γ globulin, 51.5 g/l; normal range, 20.0–30.0 g/l). Bence-Jones protein was not detected in the urine. The electromyography test showed a motor-dominant polyneuropathy with demyelinating features in the lower limbs. An ultrasonic cardiogram revealed that the interventricular septal and left ventricular posterior walls were thickened. A biopsy of the left neck lymph nodes revealed that the normal architecture was lost, except for the presence of occasional depleted follicles with concentrically arranged follicular dendritic cells, and that the architecture was effaced by polymorphic infiltrate with marked vascular proliferation (Fig. 1). Immunohistochemistry revealed the following results: CD21 (++), CD3 (+), CD4 (+), CD45RO (++), CD8 (+), Ki-67 (++; 60%), CD79a (−), anaplastic lymphoma kinase (−) and CD20 (−). The staining scores were defined as follows: −, negative (<3% cells positively stained); +, weakly positive (3–24% cells positively stained); ++, moderately positive (25–49% cells positively stained); +++, strongly positive (≥50% cells positively stained). Computed tomography scans (Fig. 2) revealed swelling of the axillary, mediastinal and retroperitoneal lymph nodes, and hepatosplenomegaly. Magnetic resonance imaging of the head was normal. Abnormal cells were confirmed by bone marrow punctures. These findings were consistent with a diagnosis of stage IV AITL, according to the World Health Organization classification (4). The patient was also diagnosed with POEMS syndrome, as determined by the criteria defined by Dispenzieri *et al* (5). Following two courses of gemcitabine (1,600 mg, days 1 and 8), oxaliplatin (150 mg, day 1), DXM (10 mg, days 1–5) and L-asparaginasum (1,000 IU, days 1–5) therapy, in a 21-day cycle, the lymphadenopathy was reduced, and the skin changes and limb neurological symptoms improved markedly.

## Discussion

AITL is classified as a high-grade malignancy, with a range of clinical symptoms and signs, including fever, weight loss, anemia, hepatosplenomegaly, thrombocytopenia, lymphadenopathy and polyclonal hypergammaglobulinemia (6). AITL mainly affects the elderly population (median age, 59–65 years), with a slight predominance in males. Geographically, the disease occurs more often in Europe compared with North America or Asia (7).

Anthracycline-based chemotherapy regimens are currently used as the first-line treatment for AITL, however, the results are mostly short term and associated with early relapse (8,9). For young patients or those suffering from relapse, studies have shown that high-dose chemotherapy combined with autologous stem cell transplantation has a higher response rate and a lower recurrence rate (10,11). Recent studies have used several chemotherapeutic agents, including pralatrexate (12), bendamustine (13), bortezomib (13) and immunomodulators such as thalidomide (14) and cyclosporin-A (15), for the treatment of AITL, both as single agents and in combination. Certain targeted drugs, such as alemtuzumab (16,17) and bevacizumab (18), also prevail in the treatment of AITL.

The overall prognosis of AITL is poor and is associated with a five-year survival rate of 30–36% and a median survival time of less than three years. Poor prognosis factors of AITL comprise an age of >60 years, a performance status of >2, more than one extranodal site, the presence of B symptoms and a platelet count of <150×10^9^/l (19).

Lymphoma with POEMS or POEMS-like syndrome is extremely rare in clinical practice, and to the best of our knowledge, only three cases have previously been reported in the literature, all of which were B-cell lymphoma (20–22). With regard to the unique manifestation of the present case, it must be determined whether a correlation exists between AITL and POEMS syndrome. We hypothesize that AITL is derived from a follicular helper T-cell subset of a germinal center; this T-cell subset promotes positive selection, proliferation and differentiation of germinal center antigen-specific B cells. A number of studies have shown that chemokine (C-X-C motif) ligand 13 (23) and CD20 (24) are highly expressed in AITL. Iqbal *et al* (25) also reported that the AITL classifier is largely reflective of the non-neoplastic cells in the microenvironment, with a significant contribution by B cells. Therefore, we postulate that T cells overactivate B cells and B cells react to T cells in the pathogenesis of AITL. This hypothesis also provides a rationale to explain the symptoms linked to B-lymphocyte activation, such as the presence of plasmacytic infiltrate in tumor biopsies and the development of hypergammaglobulinemia, as well as the manifestations of immunological dysfunction. When considering B-cell hyperstimulation stigmata, like POEMS syndrome, and the putative feeder role of B cells for neoplastic T cells, we further hypothesize that the disruption of putative B cell-T cell interactions by rituximab could improve the clinical outcome. The use of anti-CD20 therapy, such as of rituximab (26), in AITL has also been reported, but remains under debate.

In summary, this study is the first to report a case of AITL with POEMS syndrome, which may be associated with B cell-T cell interactions. The findings in this case suggest that the aberrant clones of B cells can also be caused by AITL. Therefore, clinicians should be aware of the possibility of POEMS syndrome in AITL patients, particularly when associated with polyneuropathy or endocrine alterations. Additionally, further investigation may be warranted into the use of a rituximab combination treatment to improve the clinical outcome.
